# Acute superior mesenteric artery syndrome with complete foregut obstruction following Nissen fundoplication

**DOI:** 10.1016/j.ijscr.2023.107958

**Published:** 2023-03-04

**Authors:** Donata Vaiciunaite, Inanc S. Sarici, Sven E. Eriksson, Shahin Ayazi, Blair A. Jobe

**Affiliations:** a Esophageal Institute, Department of Surgery, Allegheny Health Network, Pittsburgh, PA, United States; b Department of Surgery, Drexel University, Philadelphia, PA, United States

**Keywords:** Superior mesenteric artery syndrome (SMAS), Nissen fundoplication, Small bowel obstruction, Multiple sclerosis (MS)

## Abstract

**Introduction and importance:**

Superior mesenteric artery syndrome (SMAS) is a rare but severe condition characterized by acute angulation of the aortomesenteric axis. It can result in compression and obstruction of the third part of the duodenum leading to life-threatening dilation and perforation of the proximal duodenum and stomach.

**Presentation of case:**

We report a rare case of a patient with postural abnormality secondary to multiple sclerosis and a borderline but normal aortomesenteric axis who developed SMAS following a paraesophageal hernia repair with Nissen fundoplication complicated by massive gastric dilation and perforation secondary due to a closed-loop-like foregut obstruction. The patient was managed with emergent damage control surgery and washout with delayed duodenojejunostomy for SMAS.

**Clinical discussion:**

SMAS with partial obstruction can mimic common complications after Nissen fundoplication such as gas-bloat syndrome. SMAS with complete obstruction is a life-threatening surgical emergency. Postoperative weight loss, large hiatal hernia reduction, gas-bloat syndrome and postural changes in this patient may have contributed to an altered aortomesenteric axis and promoted the development of SMAS. Identifying possible predisposing factors should heighten vigilance and prompt radiological evaluation and surgical management to prevent life-threatening complications.

**Conclusion:**

SMAS after Nissen fundoplication is a potentially life-threatening complication that presents with non-specific symptoms mimicking common complications like gas-bloat syndrome. A high index of suspicious should prompt early radiological evaluation in patients with predisposing factors.

## Highlights

• Superior mesenteric artery syndrome (SMAS) develops when the aortomesentic angle is narrowed, causing duodenal obstruction.
• Patients with pronounced postural abnormalities are at risk for SMAS.
• Postoperative changes after Nissen may alter aortomesenteric anatomy and provoke SMAS in at risk patients.
• Symptoms of SMAS with early or partial obstruction are indistinguishable from gas-bloat syndrome on clinical exam.
• Risk factors for SMAS should prompt preoperative imaging and lower the threshold for postoperative imaging if indicated.

## 1 Introduction

Superior mesenteric artery syndrome (SMAS) is a rare type of mechanical small bowel obstruction caused by vascular compression of the duodenum. This condition is thought to result from a loss of the mesenteric fat pad between the superior mesenteric artery (SMA) and the aorta secondary to rapid weight loss, or mechanical changes to the aortomesenteric angle due to scoliosis or abdominal or spinal surgeries [1], [2], [3], [4]. Without this fat pad the angle between the two vessels is reduced, resulting in compression and obstruction of the third segment of the duodenum. Patients most commonly present with intermittent insidious chronic pain with partial obstruction; however, acute and severe symptoms with complete obstruction may also occur [5]. These symptoms are nonspecific; therefore, diagnosis is chiefly achieved through computerized tomography (CT) utilizing criteria the radiology literature: an aortomesenteric distance less than 8-10 mm and an aortomesenteric angle less than 22 degrees [6]. Here we report a rare case of SMAS mimicking a closed-loop stomach obstruction following a recurrent large paraesophageal hernia repair with Nissen fundoplication. This work has been reported in line with the SCARE 2020 criteria [7].

## 2 Presentation of case

**Fig. 1:**
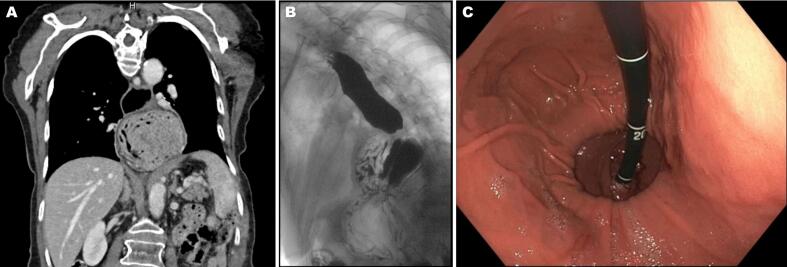
Preoperative thoracoabdominal computerized tomography (CT), barium esophagram, and endoscopic images: A) Coronal view of a contrast-enhanced CT slice demonstrates the paraesophageal hernia with a large portion of the stomach within the thoracic cavity. B) Barium swallow study shows large paraesophageal hernia with poor clearance of barium and dilated esophagus. C) Retroflex view of the gastroesophageal junction shows a 5 cm large paraesophageal hernia with a sliding component.

**Fig. 2:**
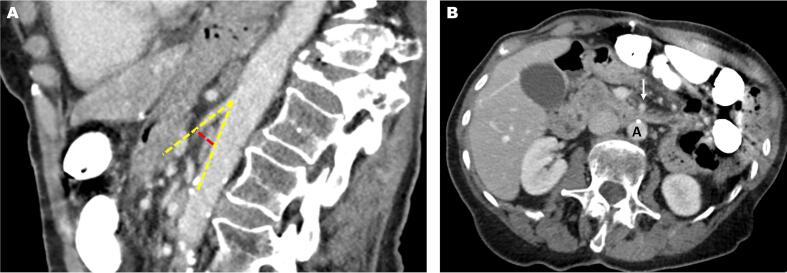
Preoperative abdominal computerized tomography (CT) images: A) Sagittal view contrast-enhanced CT shows a borderline-normal aortomesenteric with an angle of 28 degrees (dashed yellow line) and an aortomesenteric distance of 10 mm (dashed red line). B) Axial contrast-enhanced CT shows the aortomesenteric distance between superior mesenteric artery (white arrow) and aorta (A) without duodenal obstruction. (For interpretation of the references to colour in this figure legend, the reader is referred to the web version of this article.)

A 72-year-old female with a past medical history of a type IV paraesophageal hernia (PEH) status post emergent repair two years ago presented to clinic with bothersome dysphagia, chest pressure and heartburn, concerning for recurrence. Past medical history was also significant for chronic severe progressive and medically refractory multiple sclerosis. This condition had multiple levels of spinal involvement complicated by a contorted body habitus, bilateral paraparesis and leg weakness, leaving her unable to walk. She also was chronically dependent on morphine to manage the associated lower extremity muscle pain and spasm. Computer tomography (CT) was significant for a recurrent large PEH and gastric volvulus **(**Fig. 1**)**. Imaging also showed normal aortomesenteric distance of 10 mm (normal = 10-28 mm) and angle of 28 degrees (normal = 28–65°) **(**Fig. 2**)** [6]. There were no clinical symptoms of SMAS. Risks and benefits of surgery were thoroughly discussed and given the severity of her symptoms, the patient agreed to proceed with a laparoscopic repair of the recurrent PEH with Nissen fundoplication. The surgery was uneventful; performed in our practice's typical fashion. The patient was discharged home in stable condition on the second post-operative day.

**Fig. 3:**
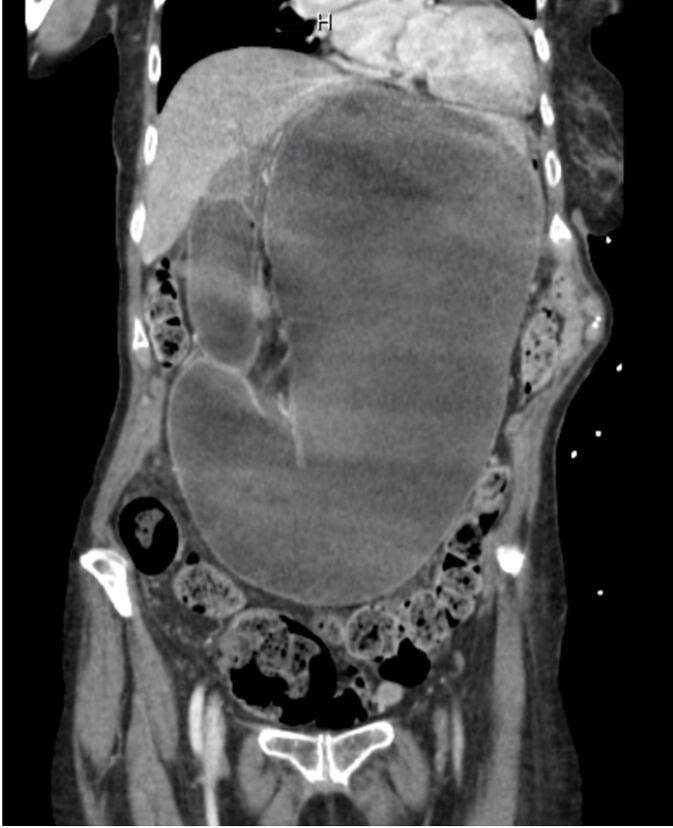
Postoperative coronal contrast-enhanced computerized tomography image demonstrates marked distension of the stomach and proximal duodenum. Transverse colon displaced to the pelvis by the distended stomach.

**Fig. 4:**
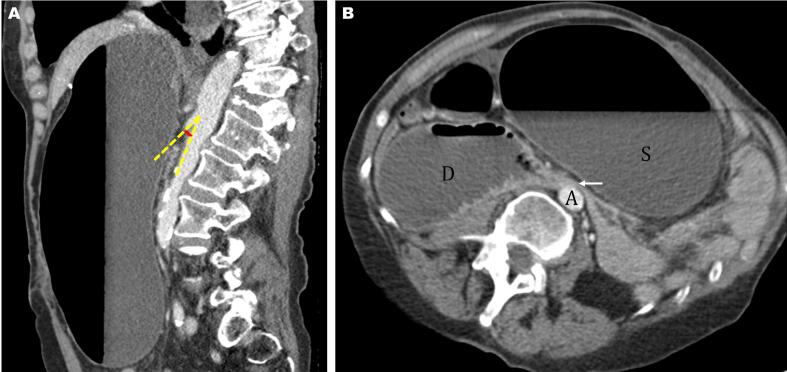
Postoperative abdominal computerized tomography (CT) images: A) Sagittal view contrast-enhanced CT shows a narrow aortomesenteric angle of 16.6 degrees (dashed yellow line) and a reduced aortomesenteric distance of 5.9 mm (red line). B) Axial view contrast-enhanced CT shows dilated stomach (S) and proximal duodenum (D), with transition point (white arrow) located at aortomesenteric level. (For interpretation of the references to colour in this figure legend, the reader is referred to the web version of this article.)

Three weeks after surgery the patient presented to the emergency department complaining of nausea, vomiting, bloating, and distention. On exam, she was hypotensive, tachycardic, and distended with a tender tympanic abdomen. Laboratory testing revealed a hypochloremic hypokalemic metabolic alkalosis of 7.48 with lactic acidosis of 10.6 mmol/L. CT scan showed severely dilated stomach and duodenum with a transition point in the third part of the duodenum as it passed between the SMA and aorta (Fig. 3). Additionally, a proximal obstruction at the esophagogastric junction due to fundoplication was noted, resulting in pronounced gastric distension mimicking a closed loop obstruction. The aortomesenteric distance and angle were decreased to 5.9 mm and 16.6°, respectively, diagnostic for SMAS (Fig. 4). The patient was taken emergently to the operating room for diagnostic laparoscopy.

**Fig. 5:**
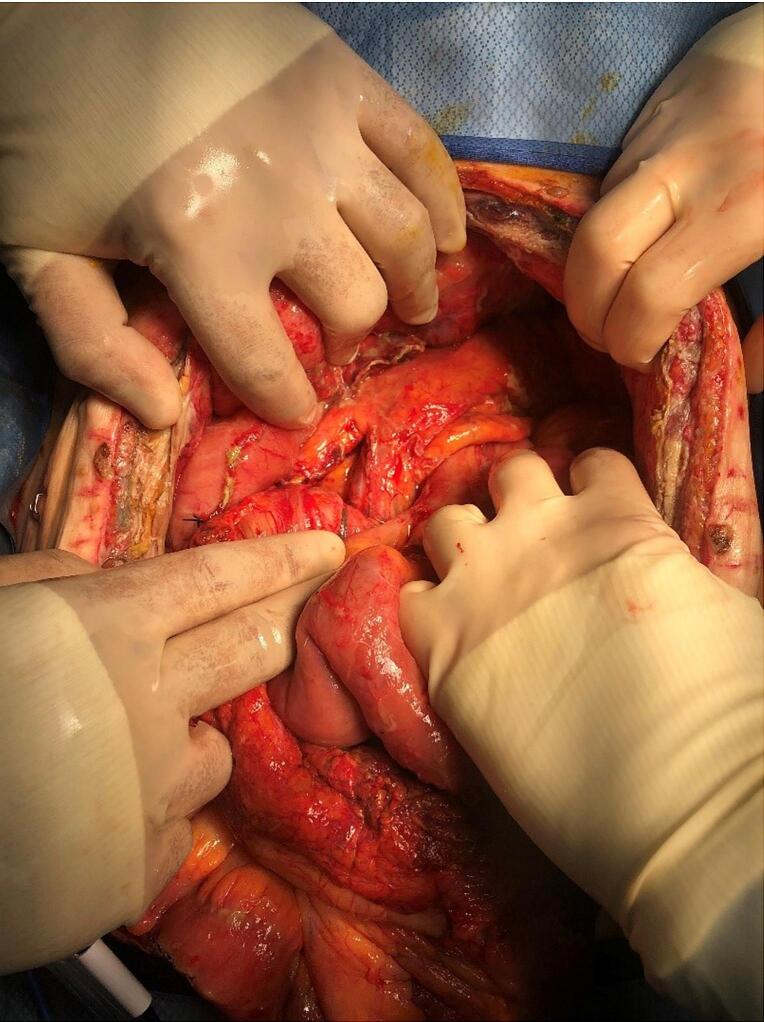
Intraoperative image shows extensive inflammation and saponification on the pancreas.

Massive gastric distention with transmural necrosis along the gastric body and free succus was noted on diagnostic laparoscopy. Surgery was converted to exploratory laparotomy and following extensive adhesiolysis a perforation was found at posterior proximal stomach. A proximal subtotal gastrectomy, abdominal washout, drain placement and feeding jejunostomy tube placement were performed. The patient remained severely hypotensive and critically ill requiring vasopressor support, therefore definitive surgery for SMAS was delayed, the open abdomen was managed by ABThera™ Open Abdomen Negative Pressure Therapy System and the patient was transferred to the surgical intensive care unit (SICU) for resuscitation and appropriate antibiosis. During the subsequent 48 h the patient's vasopressor requirement improved, but lactic acidosis did not so she was taken back to the operative room for re-exploration. Prominent necrosis of the splenic flexure and an area of the stomach proximal to the staple line were noted. Additionally, the cecum appeared dusky. A left hemicolectomy and resection of the stomach staple line were performed. The pancreas also appeared inflamed with extensive saponification **(**Fig. 5**)** and serum lipase was elevated due to reflux of pancreaticobiliary fluid secondary to the obstruction of the 3rd part of the duodenum. The patient was transferred to the SICU with an open abdomen. On re-exploration there were no more areas of necrosis and previously dusky areas appeared viable. However, the duodenum remained severely distended. Therefore, a decompressive gastroduodenal tube was placed, and a transverse colostomy was matured. Following this decompression serial lipase measurements normalized. However, the patient required another week of intensive care before she was hemodynamically stable enough for definitive surgery for SMAS syndrome. Duodenojejunostomy and esophagojejunostomy anastomosis were performed and her abdomen was closed. However, she continued to require ventilator support and underwent tracheostomy. During the following week her respiratory status did not improve, and bilious drainage was noted from the midline incision and in multiple drains. CT scan confirmed an enterocutaneous fistula. Given her comorbidities, limited mobility at baseline and these new developments her family elected to transition her comfort care.

## 3 Discussion

Superior mesenteric artery syndrome (SMAS) is a rare condition that involves compression of the third portion of the duodenum by the abdominal aorta and superior mesenteric artery. The SMA arises from the anterior surface of abdominal aorta at an acute downward angle. A retroperitoneal fat pad surrounds the SMA and supports the angle, allowing enough space for the duodenum to cross in between the two vessels. Obliteration of this fat pad by rapid weight loss decreases the aortomesenteric distance and angle, causing SMAS. The most common presentation is a partial obstruction resulting in chronic symptoms such as intermittent abdominal pain, fullness, nausea, vomiting, and weight loss [5]. Complete duodenal obstruction due to SMAS is extremely rare and presents with pronounced clinical features of upper intestinal ileus [8]. Management of complete obstruction is challenging and requires emergent surgical correction. Several predisposing factors have been identified for SMAS, including extreme weight loss, eating disorders, malignancy, trauma, neurologic disorders and surgical intervention that change the aortomesenteric angle, most commonly postural spinal surgery [2], [9], [10]. However, intraabdominal procedures also have potential to change to aortomesenteric axis anatomy [1]. Increased intraabdominal pressure and weight loss following foregut surgery can decrease the aortomesenteric angle and distance, promoting the development of SMAS and complete obstruction [11].

Nissen fundoplication is gold standard in the surgical treatment of GERD with more than 90 % of patients experiencing resolution of symptoms [12]. However, postoperative side effects are common in the short-term including dysphagia (40–70 %) and gas bloating syndrome (18–62 %) [12], [13]. Gas bloat is characterized by the inability to vent gas from the stomach due to outflow obstruction created by a tight fundoplication causing gastric dilation and a constellation of bothersome symptoms. Decreased rate of transient lower esophageal sphincter relaxation and decreased frequency of gastric belching further exacerbate this complication [13]. Compensatory anterograde peristalsis of stomach can move gastric air through small bowel, mitigating the problem. However, in the rare case of gas-bloat with an additional distal mechanical obstruction, such as SMAS, gas in the stomach becomes trapped in a closed loop-like obstruction, resulting in excessive gastric dilatation, necrosis and perforation [14].

Multiple sclerosis likely contributed to the development of SMAS in the presented case. Chronic severe MS left the patient wheelchair-bound with an abnormal vertebral alignment that altered her aortomesenteric axis alignment. Preoperative CT demonstrated thoracic kyphosis and compression deformities at the T12, L1, and L2 vertebrae, where the SMA branches off the aorta. This excessive forward curvature of the thoracic vertebrae contributed to the narrowing of the aortomesenteric distance and angle to the lower limits of normal **(**Fig. 2**)**. A similar mechanism was described in a case report of a wheelchair bound patient with MS and abnormal spinal posture who developed SMAS following a femoral fracture repair [15]. Authors suggested that the patient's abnormal spinal posture narrowed the distances between the SMA and aorta, increasing the risk for provoked SMAS. This postural component to SMAS has been most studied in patients who have undergone scoliosis surgery [16]. A study of a large cohort of patients who underwent scoliosis surgery found that patients who developed SMAS syndrome were more likely to have thoracic hyperkyphosis, similar to our patient [16]. These studies suggest that any alterations in vertebral posture may alter the retroperitoneal vascular anatomy and contribute to the development of SMAS.

There were several other predisposing factors, which may have contributed to the development of SMAS in the present case. During a hiatal hernia repair, reduction of the hernia rotates the intraabdominal anatomy down and increases intraluminal gastric tension and intraabdominal pressure, which may decrease the aortomesenteric angle. Chronic constipation secondary chronic morphine use can further increase intra-abdominal pressure. These factors can be exacerbated by gas-bloat syndrome. Studies have shown that gastric vent episodes decrease after Nissen fundoplication and that the habit of air swallowing continues for approximately 3 weeks, further increasing intraluminal gastric pressure [17]. Additionally, studies have demonstrated that multiple sclerosis can cause delayed gastric emptying, which can further increase gastric tension [18]. In concert, the compounding effect of these excessive pressures could be transmitted to the root of the SMA, narrowing the angle and leading to extrinsic compression of duodenum. As gastric pressure rises further, it becomes increasingly difficult to adequately vent through a fundoplication, similar to a proximal partial obstruction. In our patient these factors culminated in a condition that mimicked a closed loop obstruction of stomach. This obstruction leads to progressive dilation of the bowel and increased pressure, which will continue to increase until the intraabdominal pressure exceeds intravascular pressures, causing ischemia. This was the likely etiology of the perforation and necrotic and dusky areas of bowel.

Acute weight loss reduces the size of the fat pad supporting the aortomesenteric angle, another contributing factor to SMAS after Nissen in the presented case. Delayed transition to solid foods and fear of postprandial gas bloating are the most common reasons for weight loss after Nissen fundoplication. Studies have shown the average weight loss after laparoscopic Nissen fundoplication to be 3.9 kg [19]. Our patient lost 5 kg by her third postoperative week, which likely contributed to her SMAS. In a similar case, a 54-year-old female developed SMAS and gastric perforation following Nissen fundoplication, which was attributed to her 9.1 kg weight loss [11]. In a case series by Biank et al. of their 20-year experience with 22 SMAS cases, they report that 41 % of patients with SMAS had previously undergone Nissen fundoplication [20]. They also reported significant weight loss in half the cohort, with the majority of these patients having lost more than 10 % of their body weight [20]. An aortomesenteric angle less than 22° and distance less than 8 mm are diagnostic for SMAS [6]. The patient in the present case had a preoperative aortomesenteric distance of 10 mm and angle of 28°, respectively; normal, but borderline. Then she underwent surgery, and the resultant weight loss shrank the fat pad until her aortomesenteric distance and angle decreased to 5.9 mm and 16.6°, respectively.

Symptoms of bloating, nausea, inability to belch and distention after Nissen fundoplication are most commonly due to gas-bloat syndrome. However, these are also the symptoms of SMAS, making it a challenge to diagnose. The addition of abdominal pain, typical of partial obstruction of the duodenum, can also be non-specific without evidence of peritoneal irritation. Studies have shown 30 % of obstructions are misdiagnosed by senior surgeons utilizing clinical parameters [21]. The difficulty of early diagnosis can increase the likelihood of delayed presentation with complete obstruction, putting the patient at risk for life-threatening complications such as massive gastric dilation, necrosis, perforation and pancreatitis. This difficulty was highlighted in a case report by Petrosyan et al. of a patient who developed gastric perforation 7 months after Nissen and pancreatitis 2 months later, which she recovered from, but was subsequently readmitted for malnutrition, during which she was finally diagnosed with SMAS and treated [11]. Similarly, this patient's primary symptoms were daily bloating and inability to belch and vomit, most consistent with gas-bloat syndrome. The much more indolent course of disease progression compared to our case was due to transient positional relief of the obstruction and slow weight loss. Symptoms are not sufficient to diagnose SMAS. Therefore, objective diagnostic evaluations should be applied in order to prevent delayed diagnoses. Diagnostic evaluation should begin with an abdominal x-ray. However, contrast-enhanced CT scans better demonstrate the level of the obstruction and also highlight aortomesenteric anatomy. Since our patient presented with prominent symptoms of total occlusion of the duodenum and massive gastric dilation, we utilized CT scan for definitive diagnosis of SMAS. CT with contrast during preoperative GERD workup can delineate preoperative anatomy and aid in risk-assessment for SMAS for patients with predisposing factors such as postural abnormalities. Postoperatively, the development of symptoms of gas-bloat syndrome in conjunction with risk factors for SMAS, such as weight loss, should prompt further investigation.

There are various methods defined for the surgical management of SMAS. However, adequate resuscitation and hemodynamic stability should be ensured before definitive surgery. Since our patient had severe MS, which can affect sensory nerves, she presented in critical condition to emergency department with massive gastric dilation and necrosis three weeks after Nissen fundoplication. Therefore, we managed the patient with damage control approach and delayed the duodenojejunostomy until the patient was more stable.

## 4 Conclusion

Nissen fundoplication is safe and the most commonly used surgery for GERD. Superior mesenteric artery syndrome (SMAS) is extremely rare after Nissen fundoplication. Rapid weight loss, older patient, neurologic disorders and, anatomic changes in the vertebral column may predispose patients to the develop SMAS after Nissen fundoplication. Since the symptoms of gas-bloat syndrome after Nissen fundoplication and SMAS are similar clinical diagnosis is unreliable. Therefore, surgeons should be aware of the potential risk of SMAS in patients with predisposing factors such as postural disorders and appropriate preoperative diagnostic evaluation, such as a contrast CT scan, should ordered for high-risk patients. Additionally, post-operative assessment of gas-bloat syndrome should be tailored based on risk factors for SMAS to prevent life-threatening complications.

## Consent

All patients in our institution provide consent to allow use of their deidentified clinical information to be used for research purposes, case reports and scientific writings. A copy of the written consent is available for review by the Editor-in-Chief of this journal on request.

## Ethical approval

Ethical approval is exempt/waived at our institution.

## Funding

N/A.

## Guarantor

Shahin Ayazi, MD.

## Research registration number

None.

## Provenance of peer review

Not commissioned, externally peer-reviewed.

## CRediT authorship contribution statement

**Donata Vaiciunaite:** Writing – original draft, Writing – review & editing, Visualization. **Inanc S. Sarici:** Writing – review & editing, Visualization. **Sven E. Eriksson:** Writing – review & editing, Visualization. **Shahin Ayazi:** Writing – review & editing, Visualization, Supervision. **Blair A. Jobe:** Writing – review & editing, Visualization, Supervision.

## Declaration of competing interest

The authors have no relevant conflict of interest to disclose.
